# *Candida krusei* infection presenting as a right ventricular mass in a two month old Infant

**DOI:** 10.4103/0974-2069.58324

**Published:** 2009

**Authors:** Suresh V Patted, Prabhu C Halkati, Suresh T Yavagal, Ravikant Patil

**Affiliations:** Department of Cardiology, KLE University J N Medical College, Belgaum, Karnataka, India

**Keywords:** Preterm newborns, fungal infection, right ventricular mass

## Abstract

The prevalence of fungal infections in newborns and small infants is on the rise consequent to the improved care and survival of preterm babies. Most of these premature infants are immunocompromised and subjected to invasive monitoring and therapy in neonatal intensive care units making them susceptible to nosocomial infections. We report a rare case of right ventricular mass secondary to *candida krusei* infection which was excised surgically. This article reemphasizes the importance of stringent aseptic practices in neonatal intensive care units to prevent nosocomial infections and the early use of echocardiography in neonates presenting with atypical unexplained symptoms to hasten diagnosis and facilitate timely intervention.

## INTRODUCTION

With improvement in care of preterm newborns, a significant number of them survive past the immediate post natal period. This has led to an increase in the prevalence of fungal infections during infancy. Invasive monitoring with venous and arterial lines, endotracheal intubation, prolonged stay on ventilator and antibiotic usage predisposes them to fungal infections since most preterm infants are immunocompromised. In addition, some of them need steroids which make them further susceptible to these organisms.

Although risk of fungal infection in these neonates is high during their entire hospital stay, the highest risk is present in the first week of life when most of the invasive procedures are performed. Meticulous attention to asepsis, infection control, prophylaxis and aggressive treatment during this period can significantly improve the outcome in this population.

## CASE REPORT

**Figure 1:**
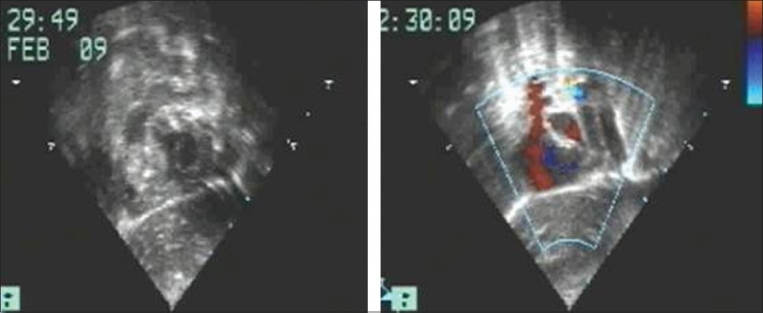
Echocardiogram in subcostal saggital view showing presence of the right ventricular mass

We present a two month old male infant, born after 34^th^ week of gestation to a healthy immunocompetent mother out of non-consanguinous marriage and delivered by a caesarian section. The baby was admitted to the NICU for respiratory distress where he received surfactant therapy for prematurity related hyaline membrane disease. He was also given IV antibiotics and needed parenteral nutrition during his hospital stay. Post discharge, the baby remained asymptomatic for eight weeks. At the beginning of the 9^th^ week mother noticed episodes of transient, loss of consciousness lasting a few seconds with spontaneous recovery. In between the episodes, the baby was active and feeding well. Beginning with three to four episodes in a day, the frequency increased and the mother noticed transient bluish discolouration of the whole body during these episodes. At this point the baby was referred to us for further evaluation and treatment. On clinical examination, the baby had mild tachypnea and tachycardia with no evidence of respiratory distress. The cardiovascular system examination was normal. Laboratory investigations revealed pancytopenia with Hb of 9.6 gm%, total white blood cell count of 1300/cu mm and platelet count of 60,000/cu mm. Serum electrolytes, blood urea, serum creatinine, serum transaminases and bilirubin were normal. In view of mild tachypnea and tachycardia, a transthoracic two dimensional echocardiography was done which revealed a large, echogenic mass with irregular borders in the right ventricle (RV). It was attached to the undersurface of the tricuspid valve and was moving across the valve into the right atrium [Figure 1].

**Figure 2:**
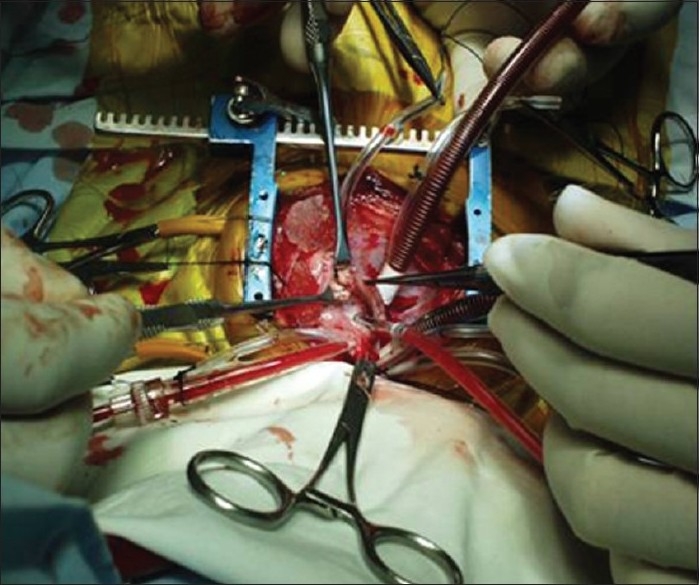
Peroperative photograph showing the right ventricular fungal ball

**Figure 3:**
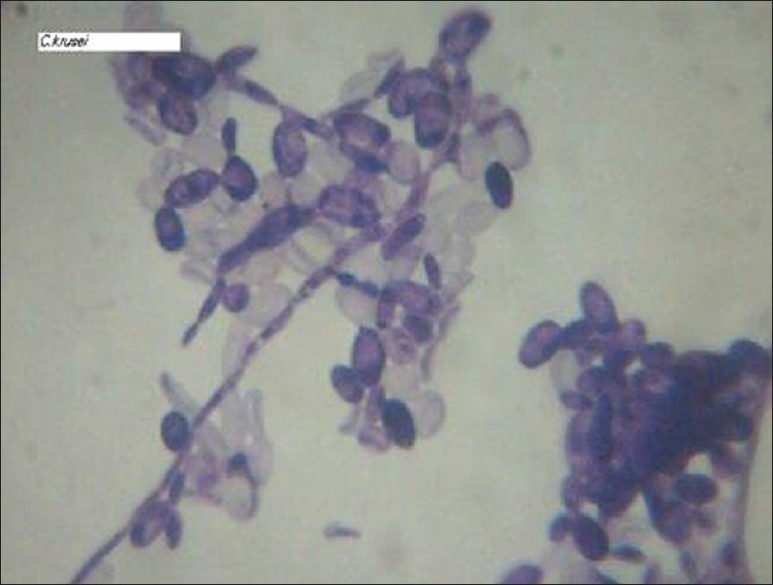
Microscopic picture of *candida krusei*, Gram stain, x 100

The baby was started on antibiotics to cover the broad spectrum of organisms along with antifungal agents. In view of his symptoms and the size of the mass, it was decided to surgically remove it. After instituting cardiopulmonary bypass, the mass was excised through a right ventriculotomy. Intra-operatively, a fragile, non-capsulated, brown coloured mass was seen attached to the undersurface of the tricuspid valve [Figure 2]. The pathological examination revealed the mass to be of fungal origin while the culture grew a rare species of *candida krusei* [Figure 3]. Subsequently patient developed necrotizing enterocolitis and disseminated fungal infection despite aggressive anti fungal therapy and succumbed on the 13^th^ post-operative day. Autopsy was not done as per the family’s wishes.

## DISCUSSION

Neonatal cardiac fungal infections occur in mostly very premature infants who are immunologically immature and compromised. Multiple invasive interventions, inadequate skin barrier to infections, poor nutrition and prolonged use of broad spectrum antibiotics that suppress protective flora are compounded by immaturity of the immune system in preterm neonates. The rate of candidemia has increased eleven-fold in the last seventeen years in our neonatal intensive care units (NICU). Candidial infection causes significant morbidity and mortality related to the disease itself as well as due to the therapy with antifungal drugs.[1]

Fungal endocarditis in neonates is usually due to candidal species. The most common strain of candia causing fungal endocarditis is *candida albicans*, but other strains like candida glabriata and *candida krusei* may rarely be responsible[2] as was seen in our case. *Candida krusei* is a rare anaerobic organism which is a normal commensal and is known to become pathologically active in immunocompromised state as was seen in this neonate. It is yeast like fungus which is urease positive.

A right sided cardiac mass in a neonate could be an uninfected thrombus, tumour or a vegetation and could present with unexplained episodes of syncope, bradycardia, apnea or hypotension secondary to intermittent showers of pulmonary emboli. These atypical symptoms in a preterm neonates with prolonged stay in the NICU with indwelling catheters and/or receiving parenteral nutrition should prompt a clinician to order an echocardiogram which can help in making the diagnosis.

Fungal endocarditis carries very high morbidity and mortality and surgery under the cover of anti-fungal therapy with Amphoterecin B (1 mg/kg/day) is the most acceptable management strategy.[3,4] We opted for surgery as the mass was big and obstructing RV inflow completely. Moreover, patient’s symptoms were highly suggestive of intermittent pulmonary embolism causing significant hemodynamic compromise. The child died despite a successful surgery due to systemic fungemia with multiorgan failure. In summary, it is important to implement best aseptic practices in the NICU to prevent fungal infection. High degree of suspicion, early diagnosis and prompt treatment with antifungals with or without surgery may help reducing morbidity and mortality associated with this infection.
